# The concentration of chromium and cobalt ions and parameters of oxidative stress in serum and their impact on clinical outcomes after metaphyseal hip arthroplasty with modular metal heads

**DOI:** 10.1186/s13018-023-03618-7

**Published:** 2023-03-21

**Authors:** Tomasz Stołtny, Michał Dobrakowski, Aleksander Augustyn, Dominika Rokicka, Sławomir Kasperczyk

**Affiliations:** 1 District Hospital of Orthopaedics and Trauma Surgery in Piekary Śląskie, Bytomska St. 62, 41-940, Piekary Śląskie, Poland; 2 Department of Biochemistry, Faculty of Medical Sciences in Zabrze, Medical University of Silesia in Katowice, Jordana St. 19, 41-808 Zabrze, Poland; 3 Department of Internal Diseases, Diabetology, and Cardiometabolic Diseases, School of Medicine With the Division of Dentistry in Zabrze, Medical University of Silesia in Katowice, Silesian Centre for Heart Diseases in Zabrze, M. Curie-Skłodowskiej St. 9, 41-800 Zabrze, Poland

**Keywords:** Metaphyseal hip arthroplasty, Modular metal heads, Metal ions, Metal-on-metal articulation, Reactive oxygen species, Oxidative stress

## Abstract

**Purpose:**

Current epidemiological data forecast an almost 40% increase in the number of hip arthroplasty performed in the population of patients with osteoarthritis in 2060, compared to year 2018. On the basis of 10 years of observation, the failure rate after a metal-on-metal hip replacement is between 56.7 and 88.9%, depending on the used implant.

**Methods:**

Seventy-six men operated using metaphyseal hip prostheses, with modular metal heads: the J&J DePuy ASR and Biomet Recap-Magnum systems, after a period of about 5–7 years after the procedure, were assessed twice (an interval of 6 months) in terms of the parameters of oxidative stress and the concentration of chromium, cobalt and ions nickel, as well as their impact on the current clinical status and quality of life.

**Results:**

The mean values of the Co and Cr ion concentrations increased in a statistically significant manner at the individual stages of the study (13.20 Co and 18.16 Cr) for J&J DePuy ASR. Using the WOMAC-hip, HHS and SF-12 rating scales, the functional status of operated patients in both study groups did not change in a statistically significant manner during subsequent visits. There was a statistically significant increase in perceived pain in patients operated bilaterally with the J&J DePuy ASR system. The severity of pain could be related to the increase in the concentration of Co and Cr ions; however, it concerned a small group of bilaterally operated patients (n = 3 + n = 4).

**Conclusions:**

Metal-on-metal configuration in hip arthroplasty significantly influences with the increase in the concentration of chromium and cobalt ions in a double assessment. A statistically significant increase in the concentration of the tested Co and Cr ions in the blood correlates with an increase in the intensity of pain, especially in patients undergoing bilateral surgery. The limitation of this study is the relatively small number of bilaterally operated patients. Elevated levels of Co and Cr ions in the blood of patients operated on with the J&J DePuy ASR system increased steadily during both follow-up visits.

## Purpose

Hip arthroplasty is currently the treatment of choice in symptomatic osteoarthritis and is therefore commonly performed in orthopedic centers around the world. Due to the dynamic epidemiological situation and the increasing population risk of hip osteoarthritis, a 37.7% increase in the frequency of hip arthroplasty in patients is forecasted in 2060, compared to 2018 [1].

With the use of metaphyseal stems, in both the valgus and varus hip, the possible individual positioning and the resulting anchorage within the metaphysis offer a wide range of reconstruction options even with abnormal femoral proximal morphology. This results in the increasing use of short metaphyseal stems in patients with complex anatomical changes or in the case of necrosis of the femoral head [2].

Low wear of friction surface, increased range of motion and a lower risk of dislocation made metal-on-metal articulation an attractive option in hip arthroplasty. Its wider use, however, has been limited in recent years due to reports of increased concentrations of metal ions and related hypersensitivity reactions in operated patients [3].

The presence of undesirable reactions to metal contaminants (ARMD) as a consequence of increased concentrations of Co and Cr ions locally and systemically is a well-described phenomenon that can accompany the presence of an implant in a metal-on-metal configuration. Mäntymäki et al. in the group of 1329 hips operated on using the Biomet ReCap-m2a-Magnum method found this negative effect in 14.3% of the respondents. ARMD worsened clinical results and aggravated pain [4].

On the other hand, during the 10-year follow-up of a group of 114 patients (122 endoprostheses) operated with an ASR XL implant, Kelly et al. reported a revision rate of 54.9%. The reason for the revision was pain (89.6%); high levels of cobalt and chromium ions (50.7%) as well as changes in the X-ray and MRI images indicate failure of arthroplasty (80.6%). All these factors were found to be present in 34.3% of the patients requiring revision [5].

Metal ions included in endoprostheses are also absorbed into the bloodstream, which can lead to systemic disorders. In addition to the aforementioned cobalt and chromium ions, trace amounts of nickel, molybdenum and tungsten alloys can also enter the circulatory system [6]. Normal blood cobalt levels for a healthy population are below 0.51 µg/l, and for optimally functioning metal-on-metal prostheses, blood cobalt levels should not exceed 2 µg/l. A malfunctioning metal-on-metal implant is reflected in concentrations higher than this value, which may lead to an increased risk of complications. The Medicines and Healthcare Products Regulatory Agency (MHRA) recommends in its guidelines the use of Co concentration in whole blood of 7 ppb (7 µg/L) in patients undergoing surgery as a screening tool for malfunctioning endoprostheses in this articulation [7].

The aim of our study was to analyze the concentrations of chromium, cobalt and nickel ions in the blood and the parameters of oxidative stress, along with the assessment of their potential impact on the postoperative clinical status, based on recognized classifications, in the population of men with coxarthrosis, after primary metal on metal hip arthroplasty using the two systems: J&J DePuy ASR and Biomet ReCap-Magnum.

## Methods

### Description of the used implants

In the first study group, consisting of 37 men, after metal-on-metal hip arthroplasty, the J&J DePuy ASR system with the Proxima stem was used. The ASR ™ acetabular component is a one-piece acetabular cup with a porous coating on its outer surface.

In 3 cases, bilateral epiphyseal arthroplasty was performed in metal-on-metal articulation with the J&J DePuy ASR system with the Proxima stem.

In the second study group, comprised of 39 men, metaphyseal hip arthroplasty with metal-on-metal articulation with the Biomet ReCap-Magnum system with a Microplasty stem was used. It consists of a pressfit acetabular cup, combined with a stem coated on its entire surface with hydroxyapatite, which is embedded in a cementless manner.

In 4 cases, a metal-on-metal bilateral arthroplasty was performed using the Biomet ReCap-Magnum system with Microplasty stem.

### Research methodology

Seventy-six men who had J&J DePuy ASR and Biomet Recap-Magnum metal modular heads implanted with metal heads 5–7 years after surgery were assessed during two follow-up visits of 6 months after each other. Results are presented as the mean of both visits.

Blood was collected at each visit for cobalt (Co), chromium (Cr), and nickel (Ni) concentrations, and clinical evaluation was performed.

Biochemical markings were conducted in the Department of Biochemistry. Faculty of Medical Sciences in Zabrze. Medical University of Silesia in Katowice, in the Laboratory of District Hospital of Orthopaedics and Trauma Surgery in Piekary Śląskie and in the Toxicology Laboratory of the EkoProfMed Medical Center in Miasteczko Śląskie.

The determinations of cobalt (Co), chromium (Cr), and nickel (Ni) concentrations were made using the atomic absorption spectrometer by Thermo Fisher Scientific, Great Britain, model ICE 3400 with a GFS35Z graphite furnace, with an automatic sample feeder, with background correction based on the transverse Zeeman effect and deuterium correction on the QuadLine lamp. The nickel concentration was determined according to Olmedo by measurement at a wavelength of 232 nm, chromium according to Huang at a wavelength of 357.9 nm and cobalt according to Schwingel Ribeiro at a wavelength of 242.5 nm. Metal concentrations are presented in μg/l [8–10].

Concentration in serum of ceruloplasmin (CER) was marked according to Richterich [11] with p-phenyldiamine reaction. Total antioxidant capacity (TAC) in serum was determined as described by Erel [12] and expressed as mmol/l. Total oxidant status (TOS) was measured in serum according to Erel [13] and expressed as μmol/l. Lipid hydroperoxides (LPH) in serum were measured according to Södergren et al. [14] using xylene orange. The results were shown in μmol/l. The concentration of lipofuscin (LPS) was determined according to the method of Tsuchida et al. [15]. The results were shown as relative units (RU) per gram of hemoglobin in erythrocytes and in RU/l in serum (the fluorescence of a 0.1 mg/ml solution of quinidine sulfate in sulfuric acid is equal to 100 RU). Superoxide dismutase (SOD) activities were measured in serum and erythrocytes according to Oyanagui [16]. The enzymatic activity of SOD was expressed in nitric units. The activity of SOD is equal to 1 nitric unit (NU) when it inhibits nitric ion production by 50%. The activities of SOD were normalized to milligrams of Hb (NU/mg Hb). Additionally, activities of isoenzymes Mn-SOD and CuZn-SOD in serum were determined. Catalase (CAT) activity in erythrocytes was measured by the kinetic method of Johansson and Håkan Borg [17]. The activity of CAT was expressed as kU/g Hb. Glutathione reductase (GR) activity in erythrocytes was measured according to Richterich [11]. The activity was expressed as *μ*moles of NADPH utilized per minute per g hemoglobin in erythrocytes (IU/g Hb). The activity of glutathione S-transferase (GST) in erythrocytes was measured according to the kinetic method of Habig and Jakoby [18], and the activity of GST was expressed as *μ*moles of thioether produced per minute per g hemoglobin in erythrocytes (mIU/g Hb). Glutathione peroxidase (GPX) activity in erythrocytes was marked using the kinetic method of Paglia and Valentine [19]. The activity of GPX was expressed as micromoles of NADPH oxidized per minute per g hemoglobin in erythrocytes (IU/g Hb).

Clinical evaluation of patients was performed based on the physical examination and using the WOMAC-HIP and HHS scale and the SF-12 quality of life scale.

WOMAC-HIP is a recognized, multidimensional disability assessment and self-assessment tool for osteoarthritis. The best result is 0 points, and the worst, 96 points [20]. The HHS scale assesses pain, function, deformity and joint mobility. A maximum of 100 points can be obtained [21]. The SF-12 scale is a proven and widely accepted tool for assessing the quality of life in terms of physical and mental health. The physical health category includes the following subscales: physical functioning, the role of physical constraints, physical pain, and general health. The mental health category includes: vitality, social functioning, the role of emotional constraints, and mental health [22].

To assess the perception of pain, the VAS scale was used, in which the measurements taken from the starting point (left end) of the scale to the mark drawn by the patient are recorded in centimeters and interpreted as their pain. The values can be used to track pain progression in a patient or to compare pain between patients with similar conditions [23].

In the physical evaluation, the length of the lower limbs was assessed twice (possible shortening or lengthening of the operated limb), and the efficiency of the hip abduction apparatus in the Trendelenburg test also was evaluated. Using a goniometer, the following mobility measurements were taken: flexion, extension, abduction, adduction, inward and outward rotational movements in the hip joint. To test the muscle strength of the quadriceps muscle of the thigh, a 5-point scale according to Lovette was used.

The study was approved by the Bioethical Committee of the Silesian Medical Chamber in Katowice under Resolution No. 9/2014 of February 24, 2014 on the medical experiment entitled: “The concentration of selected METALS and biochemical indicators in the blood and quality of life after the hip endoprosthesis surgery (ENDOMETAL project)”.

### Statistical analysis

For statistical analysis, MS Excel 2019 and Statistica 10.0 PL Software were used. The mean and standard deviation (SD) were determined for continuous variables. Shapiro–Wilk’s test was used to verify normality. Levene’s test was used to verify the homogeneity of variances. The statistical comparisons between the groups were made using a *t* test, with a separate variance or Mann–Whitney *U* test. Changes at the significance level of *p* ≤ 0.05 were considered statistically significant.

## Results

The overall clinical characteristics of the studied groups are shown in Table 1.

**Table 1:** Clinical characteristics of the studied population

Parameter	Biomet ReCap-Magnum group (n = 39)		J&J DePuy ASR group (n = 37)		*p*-value
	Mean/%	SD	Mean/%	SD	
Ischemic heart disease	8.6		0.0		0.083
Hypertension	40.0		23.5		0.146
Diabetes	8.6		2.9		0.324
Body weight (kg)	91.6	16.9	88.3	11.5	0.447
Height (m)	1.75	0.06	1.76	0.04	0.252
BMI (kg/m^2^)	30.0	4.85	28.4	3.30	0.199
Age at the time of surgery (years)	59.7	9.5	47.9	10.1	** < 0.001**
Age at the time of inspection (years)	64.7	9.46	54.6	10.01	** < 0.001**
Head size diameter (mm)	50.2	3.04	47.3	1.70	** < 0.001**
Cup size diameter (mm)	56.1	3.09	54.0	2.15	**0.002**
Local reaction (pseudotumor)	0.0		0.0		1.000

Bold values indicate *p* < 0.05

The values of the range of motion in all planes of the operated hip joint as well as the muscle strength are shown in Table 2. Significant deficiency in internal rotation in the J&J DePuy ASR group (*p* = 0.041) and a noticeable tendency in shortening of the operated limb compared to the opposite side (*p* = 0.008) were observed.

**Table 2:** Assessment of the range of mobility, muscle strength and clinical condition of the assessed hip joint

Parameter	Biomet ReCap-Magnum group		J&J DePuy ASR group		*p*-value
	Mean	SD	Mean	SD	
Flexion	106.0	12.2	110.6	13.0	0.135
Abduction	36.1	6.4	38.2	6.7	0.191
External rotation	29.7	8.2	27.4	7.3	0.212
Internal rotation	13.6	8.0	17.8	8.8	**0.041**
Adduction	29.9	6.2	31.2	6.4	0.389
HHS sum	81.3	19.4	82.1	17.6	0.858
Symmetry/Asymmetry	0.56	1.53	– 0.35	1.22	**0.008**
Lovett muscle strength	4.89	0.32	4.88	0.41	0.970

Bold values indicate *p* < 0.05

When analyzing the impact of the applied hip implant on selected elements of the patient's life, we used the WOMAC-HIP, HHS and SF-12 clinical evaluation scales. As shown in Table 3, we did not find an advantage for any of the groups in terms of patient functioning, joint mobility and deformation. In the presented study, a tendency towards increased pain in patients undergoing bilateral J&J DePuy ASR hip replacement was observed, most likely associated with an increase in Co and Cr ions (Tables 3 and 5).

**Table 3:** Clinical assessment, pain and mood assessment in the studied groups of patients

Parameter	Unilateral arthroplasty					Bilateral arthroplasty			
	Biomet ReCap-Magnum group n = 35		J&J DePuy ASR group n = 34		*p*-value	Biomet ReCap-Magnum group n = 4		J&J DePuy ASR group n = 3	
	Mean	SD	Mean	SD		Mean	SD	Mean	SD
WOMAC stiffness	1.13	1.78	1.46	1.66	0.432	1.50	1.22	2.67	1.61
WOMAC pain	3.07	4.43	3.29	3.81	0.824	4.00	3.49	4.83	1.89
WOMAC daily act	11.3	15.8	12.5	14.0	0.748	16.6	15.5	23.8	12.7
WOMAC-sum	15.5	21.7	17.2	18.9	0.728	22.1	19.6	31.3	16.2
VAS 1–10	1.67	1.90	2.01	2.19	0.489	1.63	1.31	3.00	1.00
VAS%	2.13	1.38	1.88	1.19	0.431	2.38	1.03	2.67	1.76
Number of painkillers	15.1	3.70	14.9	3.51	0.778	14.3	3.84	13.3	4.62
Hamilton-score	21.1	4.56	22.2	4.21	0.281	21.6	3.50	19.5	3.61
SF12-physical health	36.2	7.9	37.1	7.4	0.625	35.9	7.3	32.8	8.1

As dispayed in Table 4, in the J&J DePuy ASR group, compared to the Biomet ReCap-Magnum group, after unilateral arthroplasty, a higher intensity of oxidative stress was observed in the form of a higher concentration of ceruloplasmin (CER) by 12%, higher concentration of lipofuscin (LPS) in the serum by 35% and in erythrocytes by 93%. Catalase activity (KAT) was increased by 36%, GPx activity was decreased by 19% and total antioxidant status (TAC) was decreased by 9%. There were no significant differences in the oxidation-antioxidant panel between unilateral or bilateral hip arthroplasty.

**Table 4:** Oxidative stress and antioxidant enzymes in the groups of patients at particular stages of the study (data presented as mean ± SD)

Parameter	Unilateral arthroplasty					Bilateral arthroplasty			
	Biomet ReCap-Magnum group n = 35		J&J DePuy ASR group n = 34		*p*-value	Biomet ReCap-Magnum group n = 4		J&J DePuy ASR group n = 3	
	Mean	SD	Mean	SD		Mean	SD	Mean	SD
CER (mg/dl)—serum	34.8	6.81	39.0	9.60	**0.040**	32.0	3.58	42.6	13.34
TAC (mmol/l)—serum	1.06	0.16	0.96	0.16	**0.018**	1.10	0.15	1.02	0.15
TOS µmol/l—serum	7.55	3.01	9.25	9.48	0.316	6.32	2.21	13.32	7.42
LPH (μmol/l)—serum	4.04	2.07	4.83	5.26	0.415	2.82	1.23	7.92	6.34
SOD (NU/ml)—serum	18.3	1.48	18.9	2.63	0.254	18.0	1.24	17.7	1.84
MnSOD (NU/ml)—serum	10.1	0.99	11.6	3.26	0.014	10.1	1.54	10.4	1.18
CuZnSOD (NU/ml)—serum	8.18	1.40	7.32	1.87	0.034	7.95	2.35	7.28	0.91
LPS (RF)—serum	586	257	794	173	** < 0.001**	444	142	678	120
GR (IU/g Hb)—erythrocytes	8.09	1.74	8.24	1.72	0.724	9.15	1.30	7.04	1.83
KAT (kU/g Hb)—erythrocytes	413	57.5	560	62.9	** < 0.001**	431	60.6	541	106.8
SOD (NU/mgHb)—erythrocytes	162	17.9	155	12.7	0.082	147	8.14	169	18.27
GPx (IU/gHb)—erythrocytes	67.0	4.75	54.3	8.70	** < 0.001**	68.0	1.43	56.5	2.91
GST (IU/gHb)—erythrocytes	0.17	0.04	0.16	0.05	0.694	0.17	0.04	0.14	0.03
LPS (RF/gHb)—erythrocytes	725	192	1398	392	** < 0.001**	731	243	1577	298

Bold values indicate *p* < 0.05

As presented in Table 5, Cr and Co concentrations were over fivefold higher in the J&J DePuy ASR one-side hip replacement group than in the Biomet ReCap group (*p* < 0.05). In the case of patients operated bilaterally with J&J DePuy ASR, the Cr concentration was almost 5 times higher than in patients who underwent single hip arthroplasty with this implant.

**Table 5:** Concentrations of studied metal ions in the groups of patients at particular stages of the study (data presented as mean ± SD)

Parameter–mean of 2 visits	Unilateral arthroplasty						Bilateral arthroplasty			
	Biomet ReCap-Magnum group n = 35		J&J DePuy ASR group n = 34		Change %	*p*-value	Biomet ReCap-Magnum group n = 4		J&J DePuy ASR group n = 3	
	Mean	SD	Mean	SD			Mean	SD	Mean	SD
Cr concentration (μg/l)	2.04	1.31	10.45	18.16	413	**0.008**	3.99**	1.06	49.3^#^	103.04
Co concentration (μg/l)	1.58	0.93	9.26	13.20	487	**0.001**	2.98*	1.46	40.5^##^	107.68
Ni concentration (μg/l)	0.86	0.29	0.97	0.30	13	0.130	1.01	0.31	0.92	0.20
Co/Cr ratio	0.89	0.46	1.20	0.87	35	0.070	0.73	0.19	1.27	0.41

Bold values indicate *p* < 0.05

*p < 0.05 **p < 0.01 in comparison to Biomet ReCap-Magnum group

^#^*p* < 0.05 ^##^*p* < 0.01 in comparison to &J DePuy ASR group

As shown in Table 6, positive correlations were found between the elevated concentrations of chromium and cobalt ions and the increased LPS concentration in serum. Positive correlation between elevated Cr ions and CER concentration in serum was noted; also positive correlations between elevated Co ions and KAT and LPS concentration in erythrocytes were found. On the other hand, negative correlations were observed between elevated concentrations of Co and Ni and GPx concentration in erythrocytes.

**Table 6:** Correlation between studied metals and oxidative stress (Spearman R *p* < 0.05)

	Cr (μg/l)	Co (μg/l)	Ni (μg/l)
CER (mg/dl)—serum	0.24	–	–
TOS µmol/l—serum	–	–	–
LPS (RF)—serum	0.31	0.23	–
KAT (kU/g Hb)—erythrocytes	–	0.24	–
GPx (IU/gHb)—erythrocytes	–	− 0.20	− 0.32
LPS (RF/gHb)—erythrocytes	–	0.19	–

## Discussion

The motivation behind this study was the insufficient number of reports in the literature regarding the confrontation of clinical and biochemical results of various systems of metaphyseal-anchored hip endoprostheses with metal-on-metal articulation.

The algorithm for risk assessment of metal-on-metal hip replacement, proposed by Goderecci et al., uses Cr and Co concentrations in correlation with ultrasound imaged periprosthetic fluid level. A high 43.1% percentage of ASR XL implant revisions was demonstrated in the 6-year follow-up period with a sevenfold higher risk in patients with ultrasound periprosthetic fluid reservoir > 20 mm, and elevated Co and Cr concentrations. [24].

At the same time, the Chinese authors, during the 10-year follow-up period, indicate the need for revision operations in almost a quarter of the operated patients (24.3%) after cementless hip arthroplasty using the J&J DePuy ASR system. Longer follow-up of the ASR XL system correlates with a significant increase in blood concentrations of cobalt and chromium ions and deteriorating clinical outcomes and is associated with an increased risk of revision surgery [25].

Cobalt and chromium ions are released by friction between metal surfaces of metal endoprosthesis or by corrosion, which leads to the release of metal oxides, hydroxides and phosphates [6]. As a result of friction between metal surfaces, more chromium ions are released compared to cobalt ions, resulting in a low cobalt/chromium concentration ratio. And yet, when the corrosion process predominates, higher cobalt/chromium concentrations are observed. Some authors postulate that the value of this ratio exceeding 1.4 determines an increased risk of local adverse tissue reaction (ALTR) [26]. This term is defined as the inflammatory reaction of the body in the tissues surrounding the endoprosthesis, which involves the accumulation of macrophages within them and may lead to local osteolysis and loosening of the implant. [6]. In the present study, the Co/Cr concentration ratio was 0.89 in the Biomet group and 1.20 in the J&J group, which indicates the release of metal ions mainly by the mechanism of friction of metal surfaces in the Biomet group. Additionally, significantly higher concentrations of both elements, in the J&J group than in the Biomet group, suggest that the second of the used implants is more resistant to abrasion and corrosion, which may translate into a lower rate of failure of arthroplasty with its use.

Another critical factor determining the rate of wear and consequently higher failure rate of endoprostheses in the metal-on-metal configuration is the inclination angle of the acetabular component. Too steep inclination (> 55°) causes excessive load on the edge of the metal implant ("edge loading effect"), which promotes accelerated wear with the release of metal ion particles, leading to the formation of local inflammatory reactions associated with their presence along with the formation of pseudotumors [27].

As demonstrated by Guojun Jin et al., the positive correlation of the high acetabular inclination angle of the ASR XL system above 47.80° was significantly associated with elevated Cr and Co ions as well as increased pain in the operated hip joint in a group of 74 patients in the medium follow-up period over 8 years [25].

In a study by Van Der Straeten et al., operated patients were found to have higher (above the safe upper limit of normal) concentration of Co and Cr ions (4.0 for Co and 4.6 for Cr). During the first measurement, the percentage of patients with Co ions above the normal concentration was 13%, with Cr ions concentration—5%. During the second measurement, this percentage did not change [28]. The above values suggest that there is no significant increase in the Co and Cr ion concentrations for this implant placed bilaterally in a short period of observation. In our study, in patients operated on with this method, the mean concentration of Co ions was comparable—2.98, while Cr was higher—3.99, but still within the safe upper limit of normal.

Smeekes et al. showed a positive correlation between higher concentrations of selected metal ions and hip pain in operated patients due to advanced degenerative changes of the hip joint with a metal-on-metal implant. The above results are consistent with those obtained in our study**.** In addition, they also apply to the use of large-sized heads and bilateral arthroplasty in the average follow-up period of 30 months. Moreover, based on the SF36, HOOS and OHS questionnaires and radiological assessment, clinically better results were obtained in the group of patients with cobalt concentration lower than 5 μg/l. [29].

The systemic toxicity of metal ions released from endoprostheses is controversial. Many studies have shown that their concentrations in the blood often exceed the standards adopted for occupational exposure [6]. The International Agency for Research on Cancer (IARC) classifies chromium (VI) as a human carcinogen (group 1). This element occurs in several oxidation states, from 0 to + 6. It is assumed that Cr (III) does not cross the cell membrane. On the other hand, Cr (VI) has this ability, which is mutagenic. Inside the cell, Cr (VI) is reduced to the unstable forms Cr (V) and Cr (IV), eventually transforming into the stable form Cr (III). This reduction takes place with the participation of, among others, glutathione (GSH), hydrogen peroxide and ascorbate. During it, reactive oxygen species are released. [30]. Cobalt and nickel—like chromium—can also be a source of reactive oxygen species [31]. Cobalt is most often found in the + 2 and + 3 oxidation state. The International Agency for Research on Cancer (IARC) classifies this element into group 2B (possibly carcinogenic to humans). Like Cr (III), cobalt ions convert hydrogen peroxide to a hydroxide radical in the Fenton reaction [32]. In addition, cobalt displaces iron from Fe-S clusters, which are part of the respiratory chain, which disrupts its function, leading to the release of reactive oxygen species [33]. Nickel is most often found in the + 2 oxidation state. Like cobalt, it can be a source of reactive oxygen species, catalyzing the Fenton reaction [34].

An excess of reactive oxygen species can lead to an imbalance between their production and removal by the antioxidant system. This condition is known as oxidative stress. Many authors postulate that it is the basis of the toxic effects of chromium, cobalt and nickel on the human body, leading to damage to many cell structures, including cell membranes [31]. One of the markers of the intensification of oxidative stress and the related aging process is lipofuscin [35]. In this study, its concentration in serum and erythrocytes was significantly higher in the J&J group than in the Biomet group, in which lower concentrations of chromium and cobalt and significantly higher mean age are observed in parallel.

A positive correlation was also observed between the concentration of lipofuscin and the concentrations of chromium and cobalt. This observation confirms the ability of these metal ions to induce oxidative stress. It is also supported by the lower TAC value in the J&J group than in the Biomet group.

It has been proven that Cr (III) and Co (II) ions have an affinity for proteins, including antioxidant enzymes such as catalase, superoxide dismutase or glutathione peroxidase. In addition, these ions can compete for binding sites in metalloenzymes, e.g., displacing iron from catalase [32]. In this study, a negative correlation was observed between the concentration of nickel and the activity of glutathione peroxidase. The activity of this enzyme also negatively correlates with the concentration of cobalt ions. Additionally, lower glutathione peroxidase activity, than in the Biomet group, was found in the J&J group. Inverse relationships were observed in the case of catalase, the activity of which is higher in the J&J group than in the Biomet group. Additionally, the catalase activity positively correlates with the concentration of chromium ions. The higher activity of this enzyme in the group, which also has higher concentrations of chromium, cobalt and lipofuscin, can be explained by the body's adaptive defense mechanism. The higher concentration of ceruloplasmin with antioxidant properties in the J&J group, than in the Biomet group, and its positive correlation with chromium ions should be interpreted analogously. On the other hand, ceruloplasmin is also an acute phase protein [36], so an increase in its concentration may be associated with the occurrence of inflammation. The limitation of this study is the significant difference between the average age of people in the studied groups, which makes it difficult to interpret the markers of oxidative stress and the antioxidant function of the organism.

The obtained results show that the implantation of metal-on-metal metaphyseal endoprostheses is associated with an increased concentration of chromium and cobalt ions in the blood to a degree depending on the system used (J&J vs Biomet). Releasing these ions from the prosthesis results in the intensification of oxidative stress in the body and modifies the functions of the antioxidant system. However, these results should be treated with caution in terms of clinical implications, as Kovochich et al. [37] in the state-of-the-art review state that the increased risk of negative systemic effects, including cancer, related to the release of chromium and cobalt into the bloodstream, has not been proven if their concentrations do not exceed 500 and 300 µg/l of whole blood, respectively, at assumption of normal kidney function.

In our study, in the group of J&J DePuy ASR patients who underwent bilateral metal-on-metal arthroplasty with the use of a metaphyseal stem, the intensity of pain was significantly higher compared to the other groups. A relatively small group of patients undergoing bilateral surgery (n = 3 + n = 4) should be considered a limitation of this study.

The results analogous to ours were presented by Higuchi et al. in their work, assessing the clinical condition based on the HHS scale in patients after endoprosthetics of the metal-on-metal and ceramic-on-ceramic hip arthroplasty in an 8-year follow-up period. Compared to our study, the presence of lysis around the implants and the presence of pseudotumors in patients undergoing metal-on-metal hip arthroplasty were significantly more frequent, which was not reflected in the quality of life assessed by patients. [38].

The main goal of hip arthroplasty is to reduce pain, increase mobility within the joint, improve the patient's vital functions in terms of movement, and improve the patient's quality of life. Based on our research results, it seems that the use of the Biomet Recap-Magnum implant is more beneficial for the patient due to less pain, most likely associated with lower concentrations of Co and Cr ions compared to the ASR implant (on average 1.58 Co and 2.04 Cr for Biomet Recap-Magnum and an average of 13.20 Co and 18.16 Cr for J&J DePuy ASR). Due to the presented reports and the results obtained by us regarding the effectiveness and safety of metal-on-metal implants, it is recommended to periodically assess the concentration of metal ions and clinical evaluation, including the use of additional examinations (USG, MRI, CT) in patients who underwent this type of articulation. Hip replacement using the J&J DePuy ASR system results in steadily increasing concentrations of Co and Cr ions in patients during subsequent examinations, in contrast to Biomet ReCap-Magnum arthroplasty, in which the concentrations of the above-mentioned ions remain constant or decrease in the further observation period.

The limitation of our study is a small quantity of patients, especially those with a bilaterally implanted hip endoprosthesis. Therefore, it is justified to conduct further research that will allow to provide more data and broaden the knowledge on this subject.

## Conclusions

1. Both Biomet ReCap-Magnum and J&J DePuy ASR hip replacement significantly increase the concentration of chromium and cobalt ions in the blood, which, however, does not translate into pain or deterioration of clinical results. In patients undergoing bilateral hip arthroplasty using the J&J DePuy ASR system, there is an almost fivefold increase in the concentration of Cr and an over fourfold increase in the concentration of Co in the serum, compared to patients operated unilaterally.
2. Significantly elevated concentrations of Co and Cr ions in the blood of patients undergoing metal-on-metal hip arthroplasty induce oxidative stress to a degree depending on the system used and modify the function of the antioxidant system.

## Data Availability

The authors confirm that the data supporting the findings of this study are available within the article. Raw data concerning the patients are being held in the District Hospital of Orthopaedics and Trauma Surgery in Piekary Śląskie. Bytomska St. 62, 41-940 Piekary Śląskie, Poland.
